# An ^89^Zr-HDL PET Tracer Monitors Response to a CSF1R Inhibitor

**DOI:** 10.2967/jnumed.119.230466

**Published:** 2020-03

**Authors:** Christian A. Mason, Susanne Kossatz, Lukas M. Carter, Giacomo Pirovano, Christian Brand, Navjot Guru, Carlos Pérez-Medina, Jason S. Lewis, Willem J.M. Mulder, Thomas Reiner

**Affiliations:** 1 Department of Radiology, Memorial Sloan Kettering Cancer Center, New York, New York; 2 Translational and Molecular Imaging Institute, Icahn School of Medicine at Mount Sinai, New York, New York; 3 Centro Nacional de Investigaciones Cardiovasculares Carlos III, Madrid, Spain; 4 Molecular Pharmacology Program, Memorial Sloan Kettering Cancer Center, New York, New York; 5 Department of Radiology, Weill Cornell Medical College, New York, New York; 6 Department of Oncological Sciences, Icahn School of Medicine at Mount Sinai, New York, New York; 7 Laboratory of Chemical Biology, Department of Biomedical Engineering and Institute for Complex Molecular Systems, Eindhoven University of Technology, Eindhoven, The Netherlands; 8 Department of Medical Biochemistry, Amsterdam University Medical Centers, Academic Medical Center, Amsterdam, The Netherlands; 9 Chemical Biology Program, Memorial Sloan Kettering Cancer Center, New York, New York; and; 10 Center for Molecular Imaging and Nanotechnology (CMINT), Memorial Sloan Kettering Cancer Center, New York, New York

**Keywords:** immunotherapy, tumor-associated macrophages, PET/CT imaging, CSF1R inhibitor, HDL

## Abstract

The immune function within the tumor microenvironment has become a prominent therapeutic target, with tumor-associated macrophages (TAMs) playing a critical role in immune suppression. We propose an ^89^Zr-labeled high-density lipoprotein (^89^Zr-HDL) nanotracer as a means of monitoring response to immunotherapy. **Methods:** Female MMTV-PyMT mice were treated with pexidartinib, a colony-stimulating factor 1 receptor (CSF1R) inhibitor, to reduce TAM density. The accumulation of ^89^Zr-HDL within the tumor was assessed using PET/CT imaging and autoradiography, whereas TAM burden was determined using immunofluorescence. **Results:** A significant reduction in ^89^Zr-HDL accumulation was observed in PET/CT images, with 2.9% ± 0.3% and 3.7% ± 0.2% injected dose/g for the pexidartinib- and vehicle-treated mice, respectively. This reduction was corroborated *ex vivo* and correlated with decreased TAM density. **Conclusion:** These results support the potential use of ^89^Zr-HDL nanoparticles as a PET tracer to quickly monitor the response to CSF1R inhibitors and other therapeutic strategies targeting TAMs.

Breast cancer is the second leading cause of cancer-related death for women in the United States. Mortality in breast cancer results from the formation of metastases in organs such as the brain, liver, lungs, and bone marrow (1). Although most patients do not present with metastatic lesions in distant tissue on initial diagnosis, 1 in 3 women with node-negative and an even larger percentage of those with node-positive breast cancer will eventually develop distant metastases (2). The standard of care for early- and late-stage breast cancers includes chemotherapy, radiation therapy, or endocrine therapy (3). Although the 5-y survival rate for breast cancer is near 90%, the rate significantly decreases to 30% in metastatic breast cancer (4). An appreciable amount of research has described the critical roles that the innate and adaptive immune system plays in cancer development and progression (5,6). Immune checkpoint blockade, engineered chimeric antigen receptor T cells, and other drugs designed to modulate the activity and migration of innate and adaptive immune cells have become focal points of preclinical and clinical research (7).

The recruitment of myeloid cells, in particular macrophages, is considered to be one of the earliest and most crucial phases in the development of metastatic lesions (2). Macrophages exhibit dual functionality in modulating immune response: classically activated macrophages are proinflammatory, whereas alternatively activated macrophages secrete cytokines that induce tissue repair and suppress immune function (8). The accumulation of macrophages within the tumor and their shift toward an alternatively activated phenotype results in signaling cascades that induce angiogenesis, alter the extracellular matrix, and suppress adaptive immune response (9–11). Therefore, tumor-associated macrophage (TAM) burden has been correlated with rapid tumor growth, metastatic potential, and poor patient prognosis (12).

Colony-stimulating factor 1 (CSF1) and its respective receptor (CSF1R) play a key role in the recruitment and activation of macrophages (13). There are numerous clinical trials exploring the use of CSF1R inhibitors as a monotherapy or in combination with other therapeutic strategies in various types of cancer (14). Pexidartinib (PLX3397), a CSF1R inhibitor currently in phase 3 clinical trials, has shown promising results as a monotherapy and in combination with immune checkpoint therapies (14). However, traditional methods of evaluating patient response can provide misleading results. Thus, physicians require approximately 3–4 mo to properly assess treatment efficacy in patients undergoing immunotherapies (15).

The affinity of high-density lipoprotein (HDL) particles for macrophages has been well documented, and numerous formulations have been engineered to noninvasively visualize macrophage accumulation in a variety of inflammatory diseases such as atherosclerosis, arthritis, and cancers using different imaging modalities (16–20). Here, we explore the use of ^89^Zr-labeled reconstituted HDL as a macrophage-targeted diagnostic tool that might help clinicians more quickly and accurately assess the response to anti-TAM immunotherapies. To evaluate the ability of ^89^Zr-HDL to noninvasively monitor TAM burden by PET, we chose an aggressive transgenic mouse model of mammary adenocarcinoma: MMTV-PyMT (21). The biodistribution of ^89^Zr-HDL nanoparticles, specifically the accumulation in the tumor, was analyzed and compared in pexidartinib-treated and untreated MMTV-PyMT mice (Supplemental Fig. 1; supplemental materials are available at http://jnm.snmjournals.org).

## RESULTS

### Synthesis and Radiolabeling of HDL Nanoparticles

**FIGURE 1.**
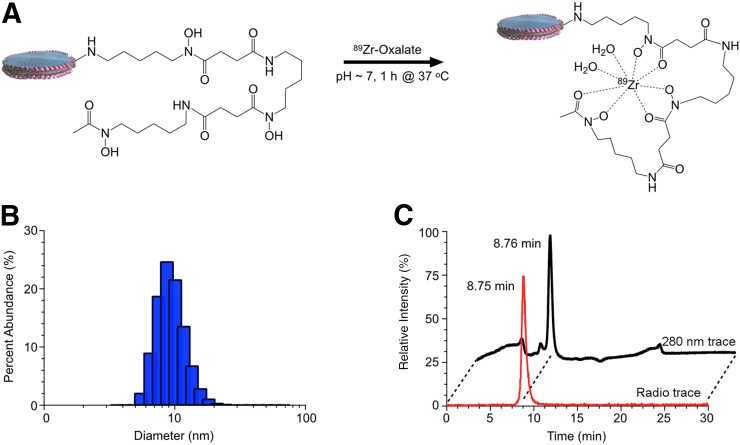
Synthesis and characterization of ^89^Zr-HDL nanoparticles. (A) Radiolabeling synthesis conditions for formation of ^89^Zr-HDL nanoparticles. (B) Dynamic light scattering analysis of HDL nanoparticles exposed to labeling conditions. (C) Radio–high-performance liquid chromatography analysis illustrating chemical and radiochemical purity of ^89^Zr-HDL nanoparticles.

^89^Zr-labeled HDL nanoparticles were prepared following our previously reported methods (Fig. 1A) (18). Deferoxamine-HDL nanoparticles had a hydrodynamic diameter of 10.9 ± 2.8 nm (*n* = 3), as determined by DLS (Fig. 1B). ^89^Zr-HDL nanoparticles were isolated in a 93% ± 6% (*n* = 3) radiochemical yield and greater than 99% radiochemical purity, as determined by radio–high-performance liquid chromatography (Fig. 1C).

### PET/CT Imaging and *In Vivo* Quantification of ^89^Zr-HDL Nanoparticles

**FIGURE 2.**
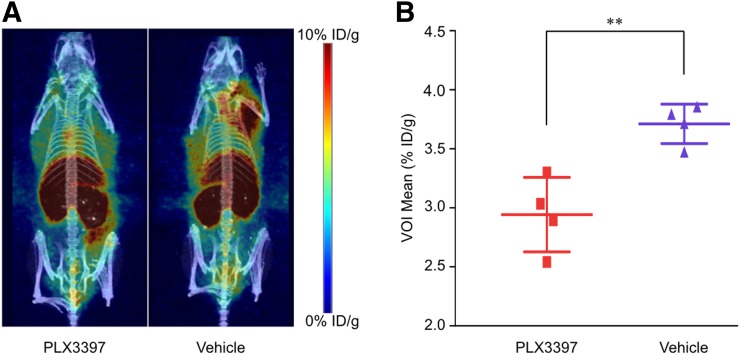
*In vivo* analysis of ^89^Zr-HDL nanoparticles, visualized by PET/CT, showing reduced accumulation in mice treated with PLX3397. (A) PET/CT images representing ^89^Zr-HDL nanoparticle distribution. (B) Quantification of ^89^Zr-HDL nanoparticle uptake determined by drawing volume of interest (VOI) over entire tumor burden. ID = injected dose. **P < 0.01.

To assess the ability of the ^89^Zr-HDL nanoparticles to act as a macrophage tracer to monitor CSF1R inhibition, MMTV-PyMT mice were treated with PLX3397 or vehicle for 5 d via daily oral gavage. This particular strain of mice begins to spontaneously develop mammary adenocarcinomas as early as 3 wk old (21). At approximately 11 wk of age, when the mammary tumors had grown to an average volume of 200 mm^3^, the treatment was initiated. PLX3397 and vehicle were administered for 5 d based on previously reported results (13). The mice were then given the ^89^Zr-HDL nanoparticles and imaged 24 h after injection. ^89^Zr-HDL uptake in tumors, determined noninvasively by drawing volumes of interest over the entire tumor mass within the mammary glands on the PET/CT images (Supplemental Fig. 2), was significantly lower in PLX3397-treated mice than in controls (2.9% ± 0.3% vs. 3.7% ± 0.2% injected dose/g, *P* < 0.01; Fig. 2B).

### *Ex Vivo* Quantification of ^89^Zr-HDL Nanoparticles and Macrophage Burden

**FIGURE 3.**
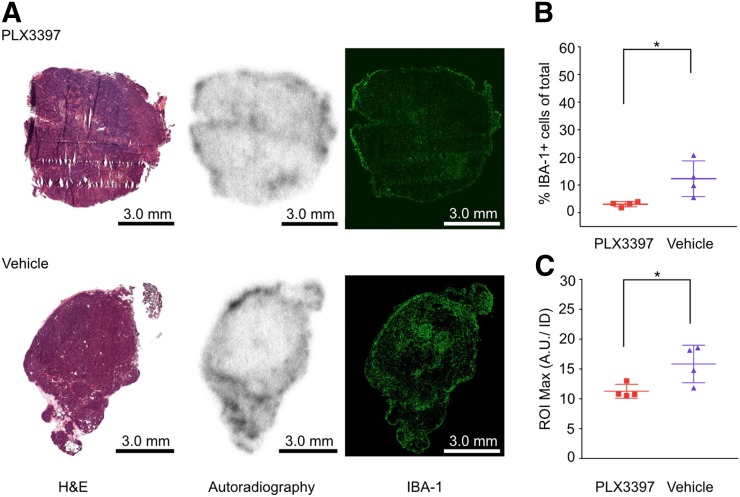
*Ex vivo* quantification of ^89^Zr-HDL nanoparticles correlates with tumor-associated macrophage density. (A) Hematoxylin and eosin (H&E), autoradiography, and immunofluorescence images of tumors excised from MMTV-PyMT mice. (B) Quantification of TAMs represented as percentage of total cells. (C) Maximum accumulation of ^89^Zr-HDL nanoparticles within region of interest (ROI) on autoradiography; activity was normalized to injected dose for each mouse. A.U./ID = arbitrary unit per injected dose. *P < 0.05.

The mice were euthanized by CO_2_ asphyxiation immediately after PET/CT imaging, and the tumors were harvested, frozen in optimal-cutting-temperature compound, and sectioned for immunofluorescence and autoradiographic analysis. Macrophage burden was quantified through IBA-1 staining followed by immunofluorescence imaging, with observed macrophage densities of 3.1% ± 0.9% and 12.3% ± 6.4% IBA-1 positive cells for PLX3397- and vehicle-treated mice, respectively (*P* < 0.05; Fig. 3B). In addition, the ^89^Zr-HDL nanoparticle accumulation was assessed using autoradiography and normalized to the injected dose. This analysis showed a significantly lower radioactivity deposition in tumors from PLX3397-treated mice than in controls (11.3 ± 1.2 vs. 15.8 ± 3.2 maximum arbitrary unit per injected dose, *P* < 0.05; Fig. 3C). The differences in nanoparticle accumulation, as observed in both *in vivo* and *ex vivo* analyses, correlate with the changes in TAM density as a result of CSF1R inhibition (22,23).

## DISCUSSION

The purpose of this study was to evaluate the use of an ^89^Zr-HDL nanoparticle as a macrophage tracer that might provide clinicians an additional tool to assess the effect of CSF1R inhibitors and other immunotherapies on TAMs. The intricate role TAMs play in altering immune function within the tumor microenvironment has made them of particular interest as targets in numerous therapeutic studies (11,14). The combination of therapeutic strategies altering both the innate and the adaptive immune system is promising; however, there are currently no accepted biomarkers that can accurately predict immunotherapy efficacy (7). Traditional methods such as RECIST can be misleading when evaluating patients undergoing immunotherapy, because tumor size can vary with changes in immune cell infiltration (15,24,25). This phenomenon, known as pseudoprogression, prevents physicians from contemporaneously assessing therapeutic responses, delaying conclusive evaluation to at least 3–4 mo after treatment initiation (26,27). Therefore, a significant clinical need exists to develop methods that can quickly and accurately monitor the effect of these therapies on TAM populations.

The observed ^89^Zr-HDL uptake assessed through *in vivo* PET/CT and *ex vivo* autoradiography analyses showed significant differences between PLX3397- and vehicle-treated cohorts. The excised tumors included areas of high fat content, as they were collected from the mammary fat pad, making it difficult to draw regions of interest that included only the cancer cells and stroma. When the activity was averaged over the drawn region of interest, a decrease in nanoparticle accumulation as a result of PLX3397 treatment was observed (Supplemental Fig. 3B). However, the differences in accumulation between the two groups were not statistically significant. Thus, the maximum accumulated activity within each tissue section was also analyzed to reduce the impact of areas with high fat content and very low nanoparticle accumulation on the analysis. The resulting data for the excised tumors showed statistically significant differences in ^89^Zr-HDL nanoparticles between the two cohorts (Fig. 3C). The immunofluorescence analysis provided evidence of effective reduction in macrophage density within the tumor as a result of the CSF1R inhibition. In addition, whereas IBA-1 is a prominently used macrophage stain in immunofluorescence, its expression is not exclusive to macrophages (28). IBA-1 can also appear on other myeloid cells such as monocytes and some lymphocytes. CSF1R inhibition, using PLX3397, was shown to be ineffective at reducing infiltration of monocytes and dendritic cells in the MMTV-PyMT mouse model (13). Thus, any IBA-1 staining associated with these cell populations may have led to smaller observed differences in TAM populations between the PLX3397- and vehicle- treated cohorts (13). Although there was considerable variation in TAM density between tissue sections, illustrating the highly heterogeneous nature of the tumor microenvironment, the differences between the PLX3397- and vehicle-treated cohorts remained statistically significant (Fig. 3B). A correlation between macrophage density and ^89^Zr-HDL nanoparticle accumulation was observed in both the PET/CT and the *ex vivo* analyses (Supplemental Fig, 3). As is apparent in the PET/CT images, there was significant accumulation of the ^89^Zr-HDL nanoparticles within the liver (Fig. 2A). Thus, whereas the tracer is ostensibly capable of imaging tumors in most tissues, the ^89^Zr-HDL nanoparticles would likely be unable to delineate metastases within the liver. The observed differences in nanoparticle accumulation, assessed using both *in vivo* PET/CT imaging and *ex vivo* autoradiography, were the result of modulations in TAM density as a consequence of treatment with the CSF1R inhibitor, pexidartinib. As observed in the *ex vivo* analysis, the macrophage content and nanoparticle accumulation was very heterogeneous. The use of PET/CT was able to overcome this heterogeneity, as ^89^Zr-HDL nanoparticle accumulation provided a quantifiable means of evaluating TAM content over the entire tumor area. Thus, the use of ^89^Zr-HDL nanoparticles as a macrophage-avid PET tracer provides a noninvasive tool to quantitatively assess overall macrophage burden and could provide clinicians the means to quickly and accurately assess response to macrophage-targeted therapies.

## CONCLUSION

The data presented herein show that ^89^Zr-HDL tumor uptake correlates with macrophage density within the tumor microenvironment. The statistically significant modulation in TAM burden, as a result of PLX3397 treatment, could be observed by quantifiable differences in ^89^Zr-HDL nanoparticle uptake using PET imaging within 1 wk of initiating treatment. Thus, ^89^Zr-HDL nanoparticles are a promising tool that could potentially help physicians more rapidly and accurately determine the early response to therapies targeting the immunosuppressed tumor microenvironment.

## DISCLOSURE

This work was supported by the MSK Molecularly Targeted Intraoperative Imaging Fund, the Tow foundation, and National Institutes of Health grants R35 CA232130, R01 CA204441, P30 CA008748, R01 CA220234, and F32-EB025050. No other potential conflict of interest relevant to this article was reported.

**KEY POINTS**

**QUESTION:** Is it possible to utilize a macrophage-selective ^89^Zr-HDL nanoparticle as a PET imaging agent to monitor the response to a CSF1R inhibitor?
**PERTINENT FINDINGS:** In a murine breast cancer model we observed significant differences in ^89^Zr-HDL nanoparticle uptake with 3.7 ± 0.2 %ID/g and 2.9 ± 0.3 %ID/g for the vehicle- and pexidartinib-treated mice, respectively. This reduction in nanoparticle uptake correlated with decreased tumor-associated macrophages as a result of CSF1R inhibition.
**IMPLICATIONS FOR PATIENT CARE:** The results in this manuscript provide evidence supporting the possible translation of the ^89^Zr-HDL nanoparticles as a means of monitoring CSFR1 inhibition and potentially other therapeutic strategies that modulate tumor-associated macrophage activity.
