# Supplementary material for: An 89Zr-HDL PET Tracer Monitors Response to a CSF1R Inhibitor
Source: J Nucl Med. 2020 Mar;61(3):433–6. doi: 10.2967/jnumed.119.230466 (PMC7067531; doi:10.2967/jnumed.119.230466)
Supplement: Supplementary file 1 [file jnm230466SupplementaryData.pdf]

MATERIALS AND METHODS

Materials. 1-(4-Isothiocyanatophenyl)-3-[6,17-dihyroxy-7,10,18,21-tetraoxo-27-[N-acetylhydroxylamino) 6,11,17, 22 -tetraazaheptaeicosane]thiourea (DFO-p-NCS) was purchased from Macrocyclics, while phospholipid 1,2-dimyristoyl-*sn*-glycero-3-phosphocholine (DMPC) was purchased from Avanti Polar Lipids. Apolipoprotein A1 (APOA1) was collected from human plasma using a previously described protocol (*16*). Pexidartinib (PLX3397) was purchased from Selleck chemicals. All other reagents were purchased from Sigma Aldrich.

Isotope Production. The ^{89}Zr-oxalate was produced at Memorial Sloan Kettering Cancer Center (MSK) on an EBCO TR19/9 variable-beam energy cyclotron (EBCO Industries Inc.) via the ^{89}Y(p,n)^{89}Zr reaction and purified in accordance with previously reported methods to yield ^{89}Zr with a specific activity of 195−497 MBq/μg. Activity measurements were made using a Capintec CRC-15R Dose Calibrator (Capintec).

HPLC and Radio-HPLC. High-performance liquid chromatography (HPLC) was performed on a Shimadzu HPLC equipped with two LC-10AT pumps and an SPD-M10AVP photodiode array detector. Radio-HPLC was performed using a Lablogic Scan-RAM Radio-TLC/HPLC detector. Size exclusion chromatography (SEC) was performed using a Superdex 10/300 column (GE Healthcare Life Sciences) with PBS as the eluent at a flow rate of 1 mL min^{-1}.

Preparation of deferoxamine (DFO)-labeled HDL nanoparticles. HDL nanoparticles were prepared using a method previously reported (*17*). Briefly, 100 mg of DMPC was dissolved in chloroform and subsequently evaporated to form a thin lipid film. The film was rehydrated in 6 mL PBS at 35-40 ºC, before APOA1) was added to a final DMPC/APOA1 mass ratio of 2.5:1. The resulting solution was sonicated on ice for 15 minutes. Large aggregates were removed through centrifugation at 4,000 rpm for 5 min and the supernatant was filtered through a PES syringe filter (0.22 µm, 13 mm diameter, Celltreat Scientific Products). A buffer exchange was performed through spin filtration of the resulting suspension using a 10 kDa molecular weight cut-off (MWCO) Vivaspin 500 concentrator (GE Healthcare Life Sciences) at 5,000 rpm. The particles were dispersed in PBS and 0.1 M carbonate buffer (pH 9.0) was added until pH was adjusted to 8.7-9.0. DFO-p-NCS was then added in a 2:1 DFO to APOA1 mol ratio. The reaction was then incubated at 37 ºC for two hours, residual unbound DFO-p-NCS was removed through spin filtration (10 kDa MWCO) and the particles were subsequently suspended in PBS and passed through a 0.22 µm PES syringe filter. Size distribution analysis of the resulting nanoparticle suspension was measured using dynamic light scattering (DLS) on a Malvern Zetasizer (Malvern Instrument Ltd.) in triplicate, at 25 ºC allowing equilibration for 120 seconds, with a 173º angle of detection.

Radiolabeling of DFO-HDL nanoparticles. The ^{89}Zr-oxalate solution was neutralized to a pH of 7 using 1.0 M Na_2CO_3. Subsequently, 250 µL of HDL nanoparticles with an approximate concentration of 2.0 mg/mL with respect to APOA1 was added to the neutralized ^{89}Zr. The reaction solution was incubated at 37 °C for 1 hour, free ^{89}Zr was

then removed via centrifugation using a 10kDa MWCO vivaspin filtration tube. The ^{89}Zr-HDL nanoparticles were diluted in sterile PBS and filtered through a PES syringe filter (0.22 µm). A radiochemical purity of > 99% for the ^{89}Zr labeled HDL nanoparticles was determined by RadioHPLC.

Animal model. 6-8 week old female transgenic FVB/N-Tg (MMTV-PyMT)634Mul/J mice were purchased from Jackson Laboratories. Animals develop mammary fat pad tumors which metastasize to the lung. At 11-12 weeks of age, once tumors had reached approximately 5.0 mm in diameter, the mice were randomized by tumor burden and administered either PLX3397 (50 mg/kg), formulated in 0.5% (hydroxypropyl)methyl cellulose (HPMC) and 1.0% polysorbate 80, or vehicle via daily oral gavage for 5 days. Animal experiments were done in accordance with protocols approved by the Institutional Animal Care and Use Committee of Memorial Sloan Kettering Cancer Center and followed National Institutes of Health guidelines for animal care.

PET/CT imaging of ^{89}Zr-HDL nanoparticles. On the fifth day of PLX3397 or vehicle administration, mice were injected with approximately 5.55MBq of ^{89}Zr-HDL nanoparticles in 200 µL PBS solution via the lateral tail vein. Animals were anesthetized with isoflurane (Baxter Healthcare)/oxygen gas mixture (2% for induction, 1 % for maintenance), and subsequently imaged using an Inveon PET/CT scanner (Siemens Healthcare Global) at 24 hours post injection. Whole body PET static scans recording a minimum of 30 million coincident events were performed. The energy and coincidence timing windows were 350−650 keV and 3.432 ns, respectively.

Image analysis and quantification. Images were analyzed using ASIPro VMTM software (Concorde Microsystems) or 3D Slicer, version 4.10 (www.slicer.org). Quantification of tumor-associated activity was performed through a combination of manual and semi-automatic segmentation techniques in 3D Slicer used to define tumor volumes within the cervical, thoracic, and inguinal mammary glands. Mean uptake within each tumor segment was quantified in percentage of the injected dose per gram of tissue (%ID/g) (*18*). No partial volume corrections were applied.

Autoradiography. To determine intratumoral radiotracer distribution, digital autoradiography was performed by placing tissue sections in a film cassette against a phosphorimaging plate (Fujifilm BAS-MS2325; Fuji Photo Film) for approximately 24 hours at -20 °C. Phosphorimaging plates were read at a pixel resolution of 25 µm with a Typhoon 7000IP plate reader (GE Healthcare Life Sciences). Quantification was carried out using ImageJ software (*19*).

Tissue staining and microscopy. Once counted and weighed the excised tumors were embedded in optimal cutting temperature (OCT) medium, frozen in dry ice, and stored at -20 °C. The tumors were then sectioned at 10 µm thickness. Staining was carried out using the automated Discovery XT processor (Ventana Medical Systems) at the Molecular Cytology Core Facility at MSK. Stained sections were digitalized using a MIRAX Slide Scanner (3DHISTECH). Thresholding was performed on IBA-1 (green, Atto-488) and DAPI (blue) areas, the percentage of IBA-1 positive cells

compared with total cells was determined for each section using an automated script, written by the Molecular Cytology Core Facility at MSKCC, in ImageJ. The PLX3397 treatment and control groups are each represented by 4 mice, at least 5 sections for each mouse was used for quantification (total of 20 sections for each cohort).

Statistical analysis. Data was expressed as mean ± standard deviation, statistical analysis was performed on each data set using GraphPad Prism, Version 7.0e using parametric unpaired t-tests, assuming unequal standard deviations between the two cohorts. Results were considered statistically significant with P values < 0.05.

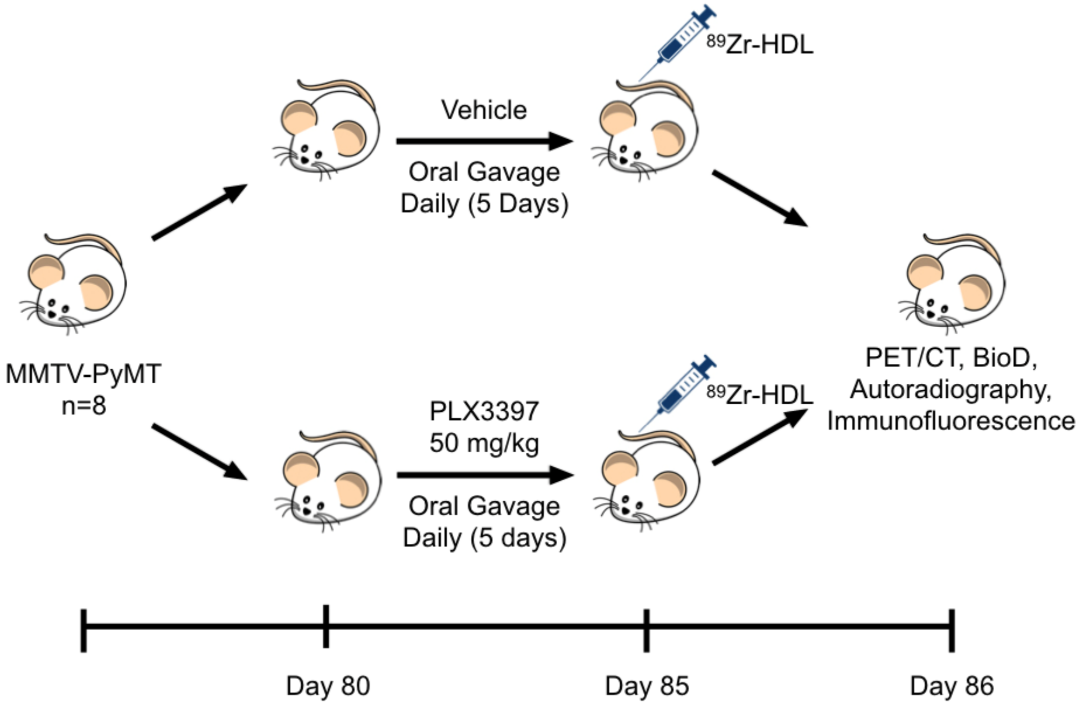

Supplemental Figure 1. Methodology for the *in vivo* assessment of ^{89}Zr-HDL nanoparticles as reporter for monitoring response to the CSF1R inhibitor, pexidartinib.

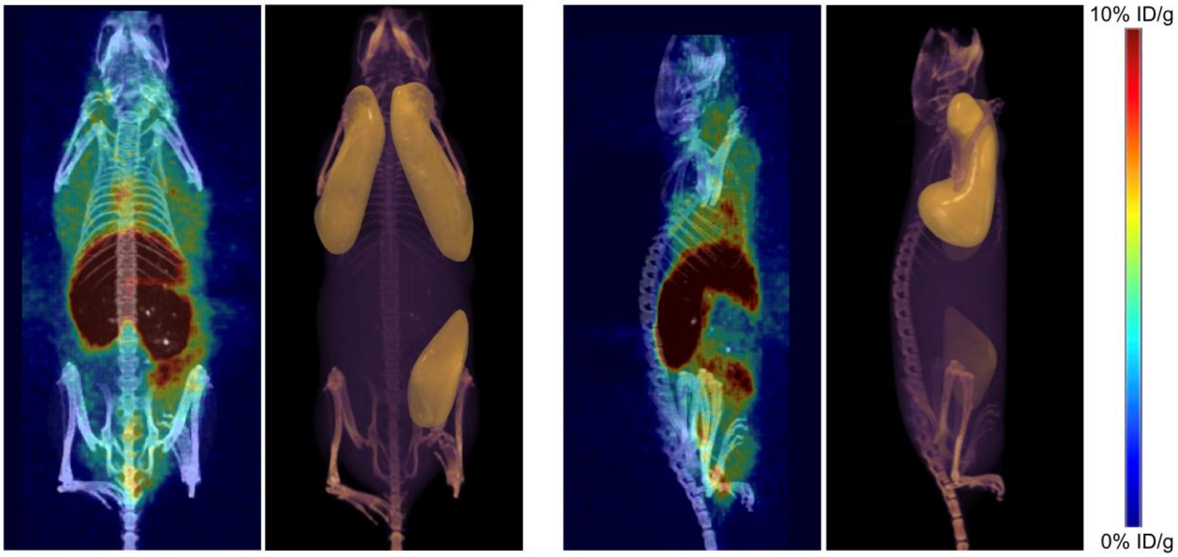

Supplemental Figure 2. PET/CT images of ^{89}Zr-HDL nanoparticles and the corresponding VOI drawn to quantify nanoparticle accumulation Quantification of ^{89}Zr-HDL nanoparticles in PET/CT images.

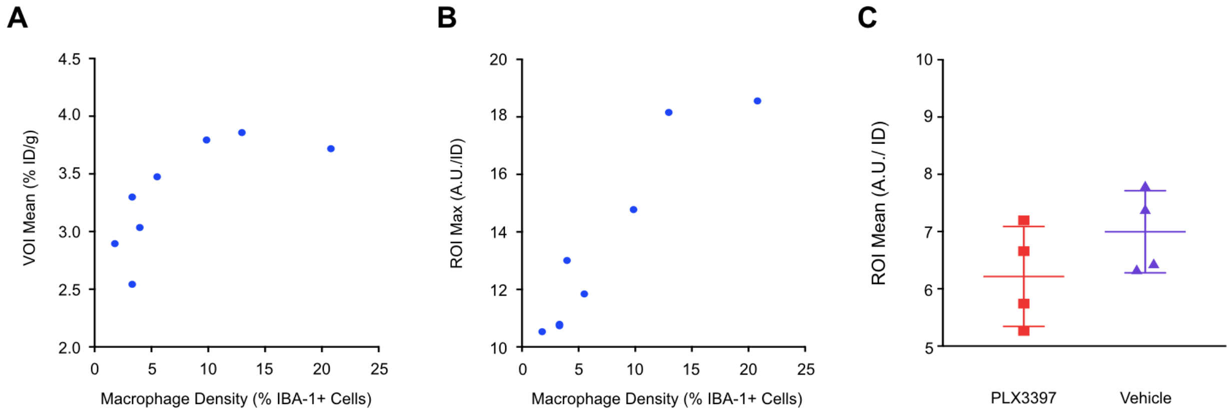

Supplemental Figure 3. Ex vivo analysis of tumor sections. A) Correlation between VOI mean from PET/CT images and macrophage density from immunofluorescence. B) Correlation between ROI max from autoradiography and macrophage density from immunofluorescence. C) Autoradiography quantification, average accumulation within ROI for tumor sections.
